# Supplementary material for: Clinical Effects of Photofunctionalization on Implant Stability and Marginal Bone Loss: Systematic Review and Meta-Analysis
Source: J Clin Med. 2022 Nov 28;11(23):7042. doi: 10.3390/jcm11237042 (PMC9739233; doi:10.3390/jcm11237042)
Supplement: Supplementary file 1 [file jcm-11-07042-s001.zip › jcm-2002255-supplementary.pdf]

Supplementary Materials

The clinical effects of photofunctionalization on implant stability and marginal bone loss: a systematic review and meta-analysis

Xinrui Lang [1], Bo Qiao [1], Ziyu Ge [1], Jiahui Yan [1], and Yanzhen Zhang [1,*]

Table S1. Search strategies

Database: Pubmed

Searched on: September 4, 2022

Results: 677

Search	Query
1	(((((((("Dental Implants"[MeSH Terms]) OR Dental implant[Title/Abstract]) OR Implant, Dental[Title/Abstract]) OR Dental Prostheses, Surgical[Title/Abstract]) OR Dental Prosthesis, Surgical[Title/Abstract]) OR Surgical Dental Prostheses[Title/Abstract]) OR Surgical Dental Prosthesis[Title/Abstract]) OR Prostheses, Surgical Dental[Title/Abstract]) OR Prosthesis, Surgical Dental[Title/Abstract])
2	(((((("Prostheses and Implants"[MeSH Terms]) OR ((Prostheses[Title/Abstract] AND Implants[Title/Abstract]))) OR Artificial Implant[Title/Abstract]) OR Artificial Implants[Title/Abstract]) OR Implant, Artificial[Title/Abstract]) OR Implants, Artificial[Title/Abstract])
3	(((((((("Dental Prosthesis, Implant-Supported"[MeSH Terms]) OR Dental Prostheses, Implant Supported[Title/Abstract]) OR Dental Prosthesis, Implant Supported[Title/Abstract]) OR Implant Supported Dental Prostheses[Title/Abstract]) OR Implant Supported Dental Prosthesis[Title/Abstract]) OR Implant Supported Denture[Title/Abstract]) OR Implant Supported Dentures[Title/Abstract]) OR Denture, Implant Supported[Title/Abstract]) OR Dentures, Implant Supported[Title/Abstract])
4	1 OR 2 OR 3
5	((((((((((((("Ultraviolet Rays"[MeSH Terms]) OR Actinic Rays[Title/Abstract]) OR Actinic Ray[Title/Abstract]) OR Ray, Actinic[Title/Abstract]) OR Ultraviolet[Title/Abstract]) OR Ultra-violet[Title/Abstract]) OR UV[Title/Abstract]) OR Functionalization[Title/Abstract]) OR Photofunctionalization[Title/Abstract]) OR Photoactivation[Title/Abstract]) OR Photo-activated[Title/Abstract]) OR Photoactivated[Title/Abstract]) OR Photofunctionalized[Title/Abstract]) OR Photofunctionalised[Title/Abstract])
6	(("Ultraviolet Therapy"[MeSH Terms]) OR Actinotherapy[Title/Abstract])
7	((((("Phototherapy"[MeSH Terms]) OR Phototherapies[Title/Abstract]) OR Photoradiation[Title/Abstract]) OR Light Therapy[Title/Abstract]) OR Light Therapies[Title/Abstract])
8	5 OR 6 OR 7
9	(((((((((((Randomized Controlled Trial[Publication Type]) OR Controlled Clinical Trial[Publication Type]) OR Randomized[Title/Abstract]) OR Randomly[Title/Abstract]) OR Trial[Title/Abstract]) OR Clinical Trial[Publication Type]) OR Groups[Title/Abstract]) OR Comparative Study[Publication Type]) OR (Control[Title/Abstract] AND Study[Title/Abstract])) OR Prospective[Title/Abstract]) OR Retrospective[Title/Abstract]) NOT ("Animals"[MeSH Terms] NOT "Humans"[MeSH Terms]))
10	4 AND 8 AND 9

Database: Embase

Searched on: September 4, 2022

Results: 129

Search	Query
1	'tooth implant'/exp OR 'dental implant':ab,ti OR ‘dental implants’:ab,ti OR ‘implant, teeth’:ab,ti OR ‘implant, tooth’:ab,ti OR ‘implants, teeth’:ab,ti OR ‘implants, tooth’:ab,ti OR ‘teeth implant’:ab,ti OR ‘teeth implants’:ab,ti OR ‘tooth implants’:ab,ti
2	'tooth implantation'/exp OR ‘dental implantation’:ab,ti
3	'single tooth implant'/exp
4	'endosseous implant'/exp
5	'implant-supported denture'/exp OR ‘dental prosthesis’:ab,ti OR ‘dental prosthesis, implant-supported’:ab,ti OR ‘implant-supported dental prostheses’:ab,ti OR ‘implant-supported dental prosthesis’:ab,ti OR ‘implant-supported dentures’:ab,ti
6	1 OR 2 OR 3 OR 4 OR 5
7	'ultraviolet radiation'/exp OR ‘ultra violet’:ab,ti OR ‘ultraviolet’:ab,ti OR ‘UV’:ab,ti
8	'functionalization'/exp OR 'functionalization':ab,ti
9	'photofunctionalization'/exp OR 'photofunctionalization':ab,ti
10	'photoactivation'/exp OR ‘activation, photo’:ab,ti OR ‘light reaction’:ab,ti OR ‘photo-activated’:ab,ti OR ‘photoactivated’:ab,ti OR ‘photofunctionalized’:ab,ti OR ‘photofunctionalised’:ab,ti
11	'ultraviolet phototherapy'/exp
12	phototherapy'/exp OR ‘light therapy’:ab,ti
13	7 OR 8 OR 9 OR 10 OR 11 OR 12
14	'randomized controlled trial'/exp OR 'randomization'/exp OR 'double blind procedure'/exp OR 'single blind procedure'/exp OR ‘random’:ab,ti
15	'clinical trial'/exp OR 'control group'/exp OR 'comparative study'/exp OR 'prospective study'/exp OR 'retrospective study'/exp OR 'case control study'/exp OR 'longitudinal study'/exp
16	‘case control’:ab,ti OR ‘cohort?’:ab,ti OR ‘follow up’:ab,ti OR ‘longitudinal’:ab,ti OR ‘retrospective$’:ab,ti OR ‘prospective’:ab,ti
17	'animal'/exp NOT 'human'/exp
18	13 OR 14 OR 15 OR 16 NOT 17
19	6 AND 13 AND 18

Database: Cochrane

Searched on: September 4, 2022

Results: 253

Search	Query
1	MeSH descriptor: [Dental Implants] explode all trees
2	'Dental Implant':ti,ab,kw OR 'Implant, Dental':ti,ab,kw OR 'Dental Prostheses, Surgical':ti,ab,kw OR 'Dental Prosthesis, Surgical':ti,ab,kw OR 'Surgical Dental Prostheses':ti,ab,kw OR 'Surgical Dental Prosthesis':ti,ab,kw OR 'Prostheses, Surgical Dental':ti,ab,kw OR 'Prosthesis, Surgical Dental':ti,ab,kw
3	MeSH descriptor: [Prostheses and Implants] explode all trees

4	'Prosthetic Implant':ti,ab,kw OR 'Implant, Prosthetic':ti,ab,kw OR ('Prostheses':ti,ab,kw AND 'Implants':ti,ab,kw) OR 'Artificial Implant':ti,ab,kw OR 'Artificial Implants':ti,ab,kw' OR 'Implant, Artificial':ti,ab,kw OR 'Implants, Artificial':ti,ab,kw
5	MeSH descriptor: [Dental Prosthesis, Implant-Supported] explode all trees
6	'Dental Prostheses, Implant Supported':ti,ab,kw OR 'Dental Prosthesis, Implant Supported':ti,ab,kw OR 'Implant Supported Dental Prostheses':ti,ab,kw OR 'Implant Supported Dental Prosthesis':ti,ab,kw OR 'Implant Supported Denture':ti,ab,kw OR 'Implant Supported Dentures':ti,ab,kw OR 'Denture, Implant Supported':ti,ab,kw OR 'Dentures, Implant Supported':ti,ab,kw
7	MeSH descriptor: [Dental Implantation] explode all trees
8	'Dental Prosthesis Implantation*':ti,ab,kw OR 'Implantation, Dental Prosthesis':ti,ab,kw OR 'Implantation, Dental':ti,ab,kw OR 'Implant Therapy, Dental':ti,ab,kw
9	1 OR 2 OR 3 OR 4 OR 5 OR 6 OR 7 OR 8
10	MeSH descriptor: [Ultraviolet Rays] explode all trees
11	'Actinic Rays':ti,ab,kw OR 'Actinic Ray':ti,ab,kw OR 'Ray, Actinic':ti,ab,kw OR 'Ultraviolet':ti,ab,kw OR 'Ultra-Violet':ti,ab,kw OR 'UV':ti,ab,kw
12	'Functionalization':ti,ab,kw OR 'Photofunctionalization':ti,ab,kw OR 'Photoactivation':ti,ab,kw OR 'Photo-activated':ti,ab,kw OR 'Photoactivated':ti,ab,kw OR 'Photoactivated':ti,ab,kw OR 'Photofunctionalized':ti,ab,kw OR 'Photofunctionalised':ti,ab,kw
13	MeSH descriptor: [Ultraviolet Therapy] explode all trees
14	'Actinotherapy':ti,ab,kw
15	MeSH descriptor: [Phototherapy] explode all trees
16	'Phototherapies':ti,ab,kw OR 'Photoradiation':ti,ab,kw OR 'Light Therapy':ti,ab,kw OR 'Light Therapies':ti,ab,kw
17	10 OR 11 OR 12 OR 13 OR 14 OR 15 OR 16
18	9 AND 17

Database: Web of Science
Searched on: September 4, 2022
Results: 729

Search	Query
1	TS=("Dental Implant*" OR "Implant*, Dental" OR "Dental Prosthes*, Surgical" OR "Surgical Dental Prosthes*" OR "Prosthes*, Surgical Dental" OR "Prosthes* and Implants" OR (Prostheses AND Implants) OR "Artificial Implant*" OR "Implant*, Artificial" OR "Dental Prosthes*, Implant-Supported" OR "Dental Prosthes*, Implant Supported" OR "Implant Supported Dental Prosthes*" OR "Implant Supported Denture*" OR "Denture*, Implant Supported")
2	TS=("Ultraviolet Rays" OR "Actinic Ray*" OR "Ray, Actinic" OR Ultraviolet OR Ultra-violet OR UV OR Functionalization OR Photofunctionalization OR Photoactivation OR Photo-activated OR Photoactivated OR Photofunctionalized OR Photofunctionalised OR "Ultraviolet Therapy" OR Actinotherapy OR Phototherap* OR Photoradiation OR "Light Therap*")
3	TS=("Randomized Controlled Trial" OR "Controlled Clinical Trial" OR Randomized OR Randomly OR Trial OR "Clinical Trial" OR Groups OR "Comparative Study" OR (Control AND Study) OR Prospective OR Retrospective NOT (Animals NOT Humans))
4	1 AND 2 AND 3

Table S2. List of studies subjected to full paper review eligibility but not included in the systematic review and the reasons for exclusion

Number	Author (Year)	References	Reasons for exclusion
1	Funato (2013) [21]	Funato, A.; Yamada, M.; Ogawa, T. Success Rate, Healing Time, and Implant Stability of Photofunctionalized Dental Implants. Int. J. Oral Maxillofac. Implants 2013, 28, 1261–1271, doi:10.11607/jomi.3263.	No control group
2	Funato (2013) [22]	Funato, A.; Ogawa, T. Photofunctionalized Dental Implants: A Case Series in Compromised Bone. Int. J. Oral Maxillofac. Implants 2013, 28, 1589–1601, doi:10.11607/jomi.3232.	Case-series
3	Suzuki (2013) [23]	Suzuki, S.; Kobayashi, H.; Ogawa, T. Implant Stability Change and Osseointegration Speed of Immediately Loaded Photofunctionalized Implants. Implant Dent. 2013, 22, 481–490, doi:10.1097/ID.0b013e31829deb62.	No control group
4	Kitajima (2016) [25]	Kitajima, H.; Ogawa, T. The Use of Photofunctionalized Implants for Low or Extremely Low Primary Stability Cases. Int. J. Oral Maxillofac. Implants 2016, 439–447, doi:10.11607/jomi.4054.	No control group
5	Hirota (2018) [36]	Hirota, M.; Ozawa, T.; Iwai, T.; Ogawa, T.; Tohnai, I. Effect of Photofunctionalization on Early Implant Failure. Int. J. Oral Maxillofac. Implants 2018, 33, 1098–1102, doi:10.11607/jomi.6541.	Studies on other outcomes
6	Hirota (2020) [26]	Hirota, M.; Ozawa, T.; Iwai, T.; Mitsudo, K.; Ogawa, T. UV-Mediated Photofunctionalization of Dental Implant: A Seven-Year Results of a Prospective Study. J. Clin. Med. 2020, 9, E2733, doi:10.3390/jcm9092733.	No control group
7	Kheur (2018) [37]	Kheur, M.; Lakha, T.; Kheur, S. A Split Mouth Study Evaluating the ISQ Trends, Radiographic Bone Loss and Implant Survival of Photofunctionalized and Untreated Dental Implants. Clin. Oral Implants Res. 2018, 29, 102–102, doi:10.1111/clr.59_13356.	Conference abstracts
8	Masuda (2018) [38]	Masuda, I. Clinical Study of Nongrafted Sinus Lift Using Titanium Plate and Screw for a Single Tooth Defect-Consideration for Cases Combined with UV-Photofunctionalization. Clin. Oral Implants Res. 2018, 29, 371–371, doi:10.1111/clr.256_13358.	Conference abstracts
